# Dynein Modifiers in *C. elegans:* Light Chains Suppress Conditional Heavy Chain Mutants

**DOI:** 10.1371/journal.pgen.0030128

**Published:** 2007-08-03

**Authors:** Sean M O'Rourke, Marc D Dorfman, J. Clayton Carter, Bruce Bowerman

**Affiliations:** Institute of Molecular Biology, University of Oregon, Eugene, Oregon, United States of America; Huntsman Cancer Institute, United States of America

## Abstract

Cytoplasmic dynein is a microtubule-dependent motor protein that functions in mitotic cells during centrosome separation, metaphase chromosome congression, anaphase spindle elongation, and chromosome segregation. Dynein is also utilized during interphase for vesicle transport and organelle positioning. While numerous cellular processes require cytoplasmic dynein, the mechanisms that target and regulate this microtubule motor remain largely unknown. By screening a conditional Caenorhabditis elegans cytoplasmic dynein heavy chain mutant at a semipermissive temperature with a genome-wide RNA interference library to reduce gene functions, we have isolated and characterized twenty dynein-specific suppressor genes. When reduced in function, these genes suppress dynein mutants but not other conditionally mutant loci, and twelve of the 20 specific suppressors do not exhibit sterile or lethal phenotypes when their function is reduced in wild-type worms. Many of the suppressor proteins, including two dynein light chains, localize to subcellular sites that overlap with those reported by others for the dynein heavy chain. Furthermore, knocking down any one of four putative dynein accessory chains suppresses the conditional heavy chain mutants, suggesting that some accessory chains negatively regulate heavy chain function. We also identified 29 additional genes that, when reduced in function, suppress conditional mutations not only in dynein but also in loci required for unrelated essential processes. In conclusion, we have identified twenty genes that in many cases are not essential themselves but are conserved and when reduced in function can suppress conditionally lethal C. elegans cytoplasmic dynein heavy chain mutants. We conclude that conserved but nonessential genes contribute to dynein function during the essential process of mitosis.

## Author Summary

Microtubules and microtubule-dependent motor proteins segregate chromosomes during mitosis and also promote cellular organization in nondividing cells. An essential motor protein complex called cytoplasmic dynein powers many aspects of microtubule-dependent transport, but it is currently unclear how dynein is regulated such that it can execute different processes. We have performed a genome-wide screen to isolate genes that are involved in dynein-dependent processes. We determined that 20 of the 49 genes we identified specifically influenced the viability of dynein mutant strains but not the viability of other C. elegans mutants. Many of the proteins that specifically influence dynein localized to subcellular sites where the dynein heavy chain has been reported by others to be found. Additionally, we identified four dynein components that appear to negatively regulate the force-generating dynein heavy chain. The identification and initial characterization of this group of genes represents a route to identify genes that are not themselves essential but do participate in essential processes.

## Introduction

The microtubule motor called cytoplasmic dynein has roles in diverse cellular processes including meiotic and mitotic spindle assembly and function, neuronal transport, and organelle positioning [1]. Cytoplasmic dynein is composed of a dimer of heavy chains (HCs), along with several accessory chains (ACs: intermediate, light intermediate, and light chains). Other dynein-interacting proteins, such as dynactin and LIS1, are likely present at substoichiometric levels and further modulate dynein function. The HCs contain both ATPase and microtubule binding activities and are sufficient for microtubule-based motility in vitro, moving toward the minus, or slow-growing, end of microtubules [2]. The dynein ACs provide cargo docking sites and often are encoded by multigene families in any one species [reviewed in 1,3]. In *C. elegans,* a single gene called *dhc-1* encodes a cytoplasmic dynein 1 HC, while 11 other genes encode five classes of predicted dynein ACs [3,4].

The early C. elegans embryo is an excellent system for investigating gene contributions for essential cellular processes, including cytoskeletal functions [5]. The C. elegans dynein HC DHC-1 is essential and required for multiple microtubule-dependent events during early embryogenesis [6–9]. Depletion of DHC-1 by RNA interference (RNAi) in early C. elegans embryos produces defects in female meiotic divisions, migration of the oocyte and sperm pronuclei after fertilization, and centrosome separation during mitotic spindle assembly [6]. Analysis of fast-acting *dhc-1* temperature-sensitive (ts) mutants has further revealed that dynein is required for chromosome congression to the metaphase plate during mitosis, as well as for mitotic spindle positioning [10].

While many requirements for cytoplasmic dynein are known, our knowledge of the molecular mechanisms that target and regulate dynein remains limited. Clearly, the multiple ACs can couple the dynein HC to particular substrates [11], including vesicles, nuclei, viruses, kinetochores, and rhodopsin [see table in 1]. However, reducing the function of only four of the eleven dynein ACs in C. elegans produces lethal phenotypes [12]. Thus, it remains unclear how ACs influence the different essential requirements for dynein. Another potential route for dynein regulation involves the phosphorylation state of the different dynein chains, which in some cases confers distinctive functional properties to the motor. While many examples of dynein phosphorylation exist, and cell cycle dependent changes in phosphorylation have been described [13–15], few if any studies have demonstrated a requirement for such modification during mitosis. Large-scale forward genetic screens have identified genes with requirements similar to those for dynein, but many of these encode core components of the microtubule cytoskeleton and few are known to directly influence dynein itself [12,16]. Genes that do influence dynein function might also have other essential roles, leading to pleiotropic mutant phenotypes that obscure their relationship to dynein [17–19]. Moreover, redundancy within the multigene dynein subunit families, and also perhaps between the different ACs, may complicate the identification of single gene requirements that are important for dynein function. Thus far, reducing the function of individual genes has not provided substantial insight into the mechanisms that regulate and mediate the many different requirements for cytoplasmic dynein during mitosis.

To identify potential regulators of cytoplasmic dynein, we have used a sensitized genetic background to conduct a genome-wide screen for modifiers of dynein function in C. elegans. Other groups have successfully used RNAi modifier screens to identify genes that function in particular pathways [20–23]; we have used RNAi to screen for genes that, when reduced in function, suppress the embryonic lethality associated with a temperature-sensitive (ts) allele of the *dhc-1* dynein HC gene. Using the *dhc-1*ts genetic background, we found 49 genes that, when depleted, suppress the partial loss of HC function. Twenty of these genes suppress conditional dynein HC mutants but not other conditional mutants with unrelated defects. Finally, we show that some of these dynein-specific suppressors encode proteins that may overlap with the dynein HC in subcellular localization.

## Results

**Figure 1:**
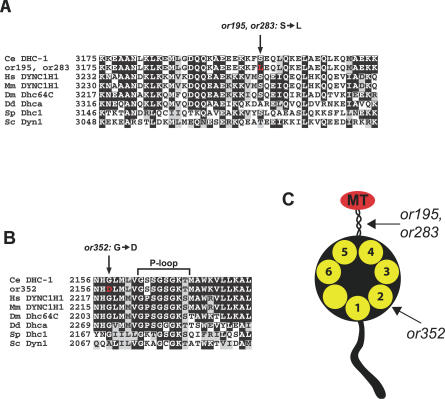
Identification of Mutations in Three Recessive *dhc-1*ts Mutant Alleles (A) *dhc-1(or195)* and *dhc-1(or283)* both have a serine changed to leucine at codon 3200, which corresponds to the N-terminal coiled-coil domain of the microtubule-binding stalk. Other metazoans and S. pombe also have a serine at this position. (B) *dhc-1(or352)* changes a glycine to an aspartic acid at codon 2158 within the Walker A region of the second AAA ATPase domain. Other organisms also have a glycine (or alanine for budding yeast) at this position. Organisms: Ce: Caenorhabditis elegans, Hs: Homo sapiens, Mm: Mus musculus, Dm: Drosophila melanogaster, Dd: Dictyostelium discoideum, Sp: Schizosaccharomyces pombe, and Sc: Saccharomyces cerevisiae. (C) Model of the dynein heavy chain and location of three ts alleles. Numbered sectors represent the six AAA domains and “MT” denotes the microtubule-binding domain.

**Figure 2:**
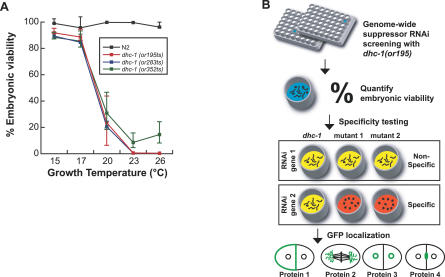
Characterization of *dhc-1*ts Mutants and Overview of the Suppressor RNAi Screening Strategy (A) Temperature versus viability plot for three recessive *dhc-1* mutants and wild-type animals. L1 larvae were grown at the indicated temperatures on L4440 control dsRNA-expressing bacterial strains and progeny were scored as viable larvae or dead embryos. (B) Screening procedure used to isolate and characterize suppressor genes. *dhc-1(or195)* mutant animals were qualitatively screened with the RNAi E. coli library to isolate suppressing genes. Embryonic viability was quantified using consistently positive E. coli clones; clones producing embryonic viability greater than 3-fold above background were kept for further analysis. We assayed the specificity of suppression by using ts mutants with defects unrelated to dynein. Finally, the localization of the suppressor proteins was determined with GFP fusion proteins.

To identify dynein suppressors, we used three different conditional and recessive *dhc-1* mutants that were identified previously [7]. These ts alleles of *dhc-1* (*or195*, *or283*, and *or352*) produce similar defects at the restrictive temperature of 26 °C, including incomplete mitotic spindle assembly in one-cell embryos and embryonic lethality [7]. We sequenced the *dhc-1* locus in the three mutants. The independently isolated *or195* and *or283* alleles each change a conserved serine to leucine at codon 3200, within the coiled-coil region of the microtubule-binding stalk domain (Figure 1A and 1C). The *or352* allele replaces a conserved glycine with aspartic acid at codon 2158, in the ATP-binding walker A motif of the second AAA ATPase domain (Figure 1B and 1C). As both missense mutations affect conserved residues, they may prove useful for engineering ts alleles in other organisms. The temperature versus viability curves of the dynein ts mutants feature a steep central transition zone ideal for modifier screening because subtle changes in temperature produce large changes in embryonic viability (Figure 2A).

To identify genes that, when reduced in function, can suppress conditional *dhc-1* mutants, we developed a high-throughput RNAi-based screen (Figure 2B). To reduce gene function we used a library of 16,757 bacterial strains that each express a dsRNA corresponding to exon-rich gene sequences [17,19]. We then tested over 99% of the bacterial strains in this library for RNAi-mediated suppression of *dhc-1(or195)* embryonic lethality at 23 °C, after raising synchronized L1 larvae to adulthood on dsRNA-expressing bacterial lawns in 48-well agar plates. This screening procedure should work to identify nonessential and essential suppressor genes, because RNAi does not always fully reduce gene function [24,25], and even if RNAi does produce lethality, cosuppression could restore viability. Nevertheless, essential genes may be missed due to earlier requirements that produce strong larval arrest, sterile, or embryonic lethal phenotypes.

**Figure 3:**
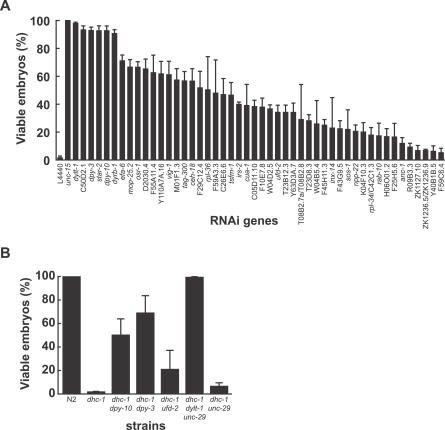
*dhc-1*-Suppressing Genes (A) Effect of reducing the function of 49 genes on embryonic viability in the *dhc-1(or195)* mutant. E. coli containing the L4440 control vector produced 1.6% viable progeny at 23 °C, while the suppressor genes produced the indicated percent viable progeny when reduced in function with RNAi. (B) Deletion and point mutation double mutants recapitulate the suppression of conditional *dhc-1* alleles observed using RNA interference. Embryonic viability of wild type, *dhc-1,* and *dhc-1* suppressor gene double mutants is displayed. The *dhc-1 unc-29* strain serves as the negative control for the *dhc-1 unc-29 dylt-1* strain.

**Figure 4:**
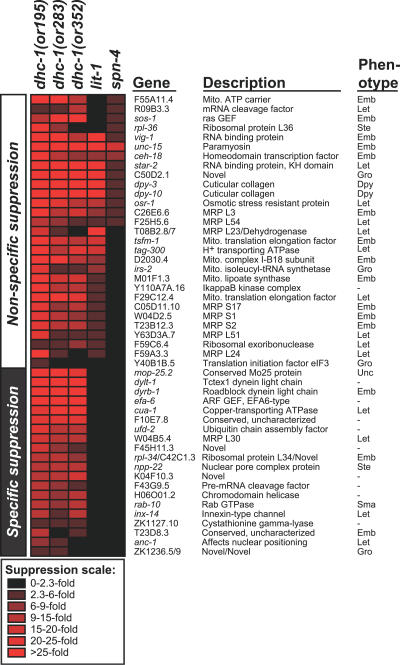
Specificity of the Dynein HC Suppressors As Assayed with Multiple ts Loci. Increasing red brightness indicates greater embryonic viability (according to the scale at bottom). Fold suppression was calculated by dividing the percent viability observed with RNAi by the background viability observed with the L4440 control, for every RNAi experiment tested in the ts mutants. Protein descriptions are from Wormbase [29]. For the numbers of progeny produced and percent viability calculation, see Table S1. Three dsRNA-producing plasmids express an RNA molecule that overlaps two different genes; this is indicated by a forward slash between the gene names. Two different gene classes are observed: specific genes only suppress multiple *dhc-1* mutant alleles while nonspecific genes suppress *lit-1* and/or *spn-4* mutants. One suppressor gene, Y40B1B.5, suppressed only one *dhc-1* allele. The phenotypes of the genes (in a wild-type or *rrf-3* background) are listed in the Phenotype column (data is from mutant or RNAi studies and collected from [29]). MRP, mitochondrial ribosomal protein, Mito, mitochondrial.

Using this screening procedure, we identified 49 bacterial clones that consistently increased embryonic viability at the semipermissive temperature. The dsRNA-producing plasmids were then sequenced to verify gene identity. Quantification of embryonic viability using *dhc-1(or195)* animals showed that the RNAi-mediated depletion of suppressor gene function increased viability to 5%–100%, compared to less than 2% in unsuppressed controls (Figure 3A; Table S1). The proteins encoded by the suppressor genes we identified are summarized in Figure 4.

As a more direct assay for dynein activity in the suppressed *dhc-1* embryos, we measured spindle length and cytokinesis success: *dhc-1* mutant embryos have severe spindle assembly defects and subsequent cytokinesis failures [6,7,10]. We shifted *dhc-1* adult hermaphrodites from 23 °C to the fully restrictive temperature of 26 °C for 3–5 hours and made time-lapse video micrographs using Nomarski optics to monitor the first embryonic cell division. This procedure results in *dhc-1(or195)* embryos with P_0_ spindles 30% the length of wild-type spindles (Figure S1). In the suppressed *dhc-1(or195)* backgrounds, spindle lengths ranged from 30%–83% of wild-type lengths (Figure S1). Similarly, cytokinesis failed in unsuppressed *dhc-1(or195)* embryos 89% of the time, but most of the suppressors rescued this phenotype (Figure S1). These results indicate that most of the suppressors influence dynein-dependent cellular processes, as expected given their ability to restore viability when reduced in function.

### Specificity of Suppression

Because RNAi can reduce the function of unintended targets (so-called “off-target effects” [26,27]), we also used available mutations in some of the suppressor genes we identified to reduce their function. We constructed double mutant strains using *dhc-1(or195)* and viable deletion alleles for two suppressor genes, *dylt-1(ok417)* and *ufd-2(tm1380),* and examined embryonic viability (Figure 3B). The deletion alleles of *dylt-1* (encoding a Tctex1-type dynein light chain), and *ufd-2* (encoding a ubiquitin conjugating enzyme) both recapitulated the suppression produced by RNAi knockdown (Figure 3B). The *dpy-3(e27)* and *dpy-10(e128)* point mutation alleles [28] also suppressed embryonic lethality in double mutants (Figure 3B). Based on this small sampling, and because RNAi in C. elegans appears to be highly gene specific in the absence of close paralogs [12,19], we conclude that many of the suppressors we have identified will prove to be suppressor locus specific. The dsRNA-expressing bacterial clones we used to deplete two of the dynein suppressors (*tag-300* and ZK1127.10) probably also knock down expression of one close paralog for each locus [29].

We next asked whether the suppressors are specific for dynein function or if their depletion more generally stabilizes ts proteins. We tested for specificity using two conditional mutants with cell fate patterning defects unrelated to dynein function, *lit-1(or131)* and *spn-4(or191)*. The *lit-1* gene encodes a MAP kinase-related protein [30], while *spn-4* encodes a protein with an RNA binding motif [31]. We found that ten of the *dhc-1*-interacting genes significantly increased embryonic viability in both *lit-1* and *spn-4* mutants, while 18 others suppressed one or the other of these two conditional mutants when reduced in function using the same RNAi protocol as that used for *dhc-1ts* mutants (Figure 4, right two columns; Table S1). Therefore, about half of the suppressors appear to act nonspecifically on multiple ts mutants to restore embryonic viability. From here on, we will refer to the suppressors that only acted on *dhc-1,* and not on *lit-1* and *spn-4* ts mutants, as dynein-specific suppressors.

Because many ts mutations exert their effect via protein assembly or unfolding mechanisms [32], suppressor genes reduced in function by RNAi might not be expected to exhibit allele specificity with most ts mutations. To determine if the dynein suppressors are either allele or strain specific, we tested the two other conditional *dhc-1* strains (containing the *or283* and *or352* alleles). Although the *or283* allele is identical to *or195,* it provides a useful control for the presence of background mutations because the two strains were isolated independently. In most cases, depletion of the dynein-specific suppressors also restored viability to the other two ts *dhc-1* alleles. Y40B1B.5, a putative translation initiation factor, suppressed only one conditional *dhc-1* strain, and we consider this as an example of a nonspecific interaction (Figure 4, left three columns; Table S1). Two dsRNAs that do not suppress *lit-1* or *spn-4* mutants produced suppression in the *dhc-1(or195)* and *dhc-1(or283)* strains, but not in the *dhc-1(or352)* strain, perhaps indicating allele specificity or variability in the RNAi treatments. We conclude that strain background differences are relatively rare, and that the majority of the suppressors are allele-independent. To summarize, we have identified 20 genes that when reduced in function specifically suppress multiple dynein ts strains but not unrelated ts loci.

### Survey of Putative Dynein Accessory Chains

**Figure 5:**
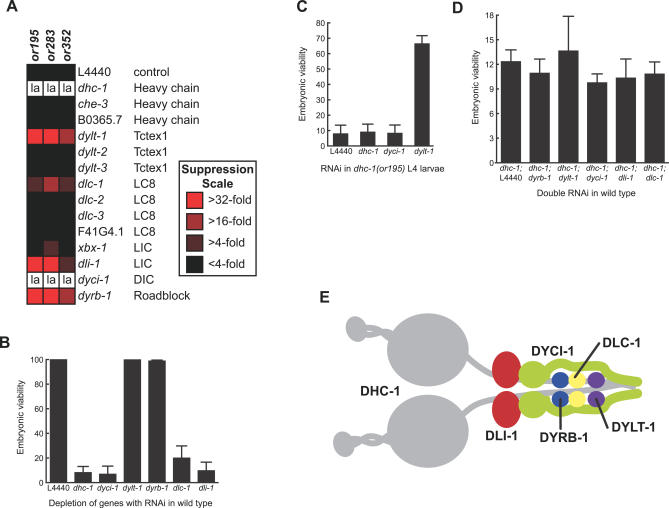
Single Members of C. elegans Dynein Subunit Families Restore Embryonic Viability When Reduced in Function in *dhc-1*ts Mutants (A) Genes encoding putative dynein subunits were tested for suppression in the three *dhc-1* backgrounds, as described in Figure 4. CHE-3 is a cytoplasmic dynein 2 HC required for retrograde transport in sensory neurons and B0365.7 encodes a highly diverged HC fragment of unknown function. The other genes are listed according to their subunit family [3]. (B) RNAi targeted towards the suppressing ACs results in variable embryonic lethality in wild-type worms. Wild-type L4 larvae were transferred to plates containing bacteria expressing the indicated dsRNAs. The observed percentage of hatching embryos is indicated. (C) Reduction of *dhc-1* or *dyci-1* function with RNAi in L4 larvae does not suppress the embryonic lethality of *dhc-1(or195)* embryos, but *dylt-1(RNAi)* does suppress embryonic lethality. (D) Codepletion of the dynein HC and dynein ACs does not result in suppression of embryonic lethality. L4 wild-type larvae were transferred to plates seeded with equal amounts of bacteria expressing *dhc-1* dsRNA and dsRNA for the second gene listed. These results correspond to the first 24 h after transferring the worms to the RNAi plates; on the second day only dead embryos were produced with all of the conditions. (E) Model of the cytoplasmic dynein protein with suppressor subunits and the DYCI-1 intermediate chain labeled [adapted from reference 33].

We were surprised to discover that depleting two predicted dynein ACs specifically suppressed the partial loss of HC function, because most dynein accessory subunits are presumed to promote dynein function by aiding dynein complex formation or mediating cargo attachment [3,11,33]. Depletion of either *dylt-1* (encoding a Tctex1-type light chain) or *dyrb-1* (encoding a roadblock-type light chain) suppressed embryonic lethality in all three ts dynein HC mutant strains (Figure 4). To extend this observation, we surveyed all genes encoding predicted dynein components for suppression of the three ts *dhc-1* mutants (Figure 5A; Table S2). We reasoned that some dynein subunit genes could have been missed in the primary screening and several dynein AC genes were not represented in the E. coli RNAi library. After using RNAi to reduce their function, we found that one of three Tctex1 homologs *(dylt-1),* one of four LC8 homologs *(dlc-1),* one of two light intermediate chains *(dli-1),* as well as the sole roadblock homolog *(dyrb-1)* each strongly suppressed the three conditional dynein mutants. Lower-level suppression was also seen for the second light intermediate chain, *xbx-1,* when its function was reduced. Thus, one gene of each of four subunit classes restores viability to the three *dhc-1* mutant strains when depleted by RNAi.

The only subunit class not found to suppress was the intermediate chain, encoded by a single gene in *C. elegans, dyci-1*. When reduced in function by RNAi, *dyci-1* produces a larval arrest phenotype like that observed for *dhc-1(RNAi);* this phenotype precludes any suppression of the conditional embryonic lethality (shown as “la” in Figure 5A). In contrast, knockdown of either *dlc-1* or *dli-1* suppresses embryonic lethality in the *dhc-1ts* mutants, even though reducing their function in otherwise wild-type embryos produces *dhc-1*-like defects, including embryonic lethality [12,34] (see Figure 5B and Discussion). The suppressing cytoplasmic dynein subunits and DYCI-1 are shown in a putative complex in Figure 5E.

We performed several genetic assays to better understand how the suppressor genes may be operating. First, suppression of *dhc-1* lethality by reducing AC function may indicate that our *dhc-1* alleles express a neomorphic and toxic DHC-1 protein: if the suppressor dynein AC subunits positively function in dynein processes, depleting them might suppress any neomorphic effects. This explanation is perhaps unlikely, because the *dhc-1*ts alleles are all recessive, but remained a possibility in *dhc-1* homozygotes. We therefore reduced dynein function using RNAi in animals that had passed through the larval arrest points for *dhc-1(RNAi)* and *dyci-1(RNAi)*. Specifically, we transferred *dhc-1(or195)* L4 hermaphrodites to plates with bacteria expressing *dhc-1* or *dyci-1* dsRNA. As control we performed *dylt-1(RNAi)* using the same procedure. We observed substantial suppression with *dylt-1* in this assay, but we saw no suppression with the heavy or intermediate chains (Figure 5C). This suggests that the DHC-1ts protein is not toxic and that *dyci-1* acts more like *dhc-1* than the other suppressing ACs because it does not suppress the heavy chain mutant.

To further examine the nature of the AC suppression, we asked if depletion of the suppressor chains could bypass the requirement for *dhc-1*. We transferred wild-type L4 larvae to plates with bacteria expressing dsRNA corresponding to both the suppressor ACs and *dhc-1*. We did not observe any suppression in these double RNAi assays (Figure 5D), suggesting that *dhc-1ts* suppression requires the residual activity of the defective DHC-1 protein. We conclude that the dynein AC suppressors inhibit or somehow oppose the function of the DHC-1ts protein, and that the *dhc-1(or195ts)* mutation does not produce a toxic gene product but simply reduces DHC-1 activity to a low, but non-null, level.

### Localization of the Dynein Suppressor Proteins

**Figure 6:**
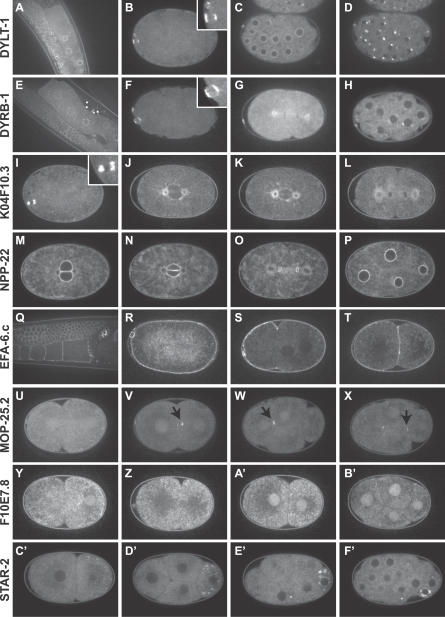
Localization of GFP-Tagged Dynein Suppressor Proteins in Wild-Type Embryos Insets show 2× magnification of meiotic spindles. (A–D) DYLT-1: oocyte nuclear envelopes (A), meiotic spindle poles (B), nuclear envelopes prior to mitosis (C), and centrosomes and mitotic spindle poles (D). Mitotic localization is most robust in embryos beyond the 12-cell stage; weaker localization to spindle poles was detectable in one- and two-cell stage embryos (see Figure 7). (E–H) DYRB-1: similar to DYLT-1, except weaker subsequent to meiosis. (I–L) K04F10.3: endoplasmic reticulum-like; meiotic spindle poles (I) and pericentrosomal during mitosis (I–L). (M–P) NPP-22: nuclear envelope and pericentrosomal (also endoplasmic reticulum-like, except for absence of meiotic spindle localization). (Q–T) EFA-6.c: nonpolarized cell cortex in oocytes and early one-cell zygotes (Q–R), anterior cell cortex in one-cell embryos subsequent to pseudocleavage (S) and present at the interface of the AB and P1 cells (T), undetectable by the four-cell stage (unpublished data). (U–X) MOP-25.2: midbody after cytokinesis (arrows indicate the spot of localization) and weak localization to spindle poles (unpublished data). (Y-B′) F10E7.8: pronuclear and nuclear (cytoplasmic signal is at least partially due to endogenous autofluorescence in this weakly expressing line). (C′–F′) STAR-2 (a nonspecific suppressor gene): apparent localization to germline P-granules.

To further explore how the suppressor proteins function, we examined the subcellular localization of nine of them as stably expressed N-terminal GFP::S fusions. We chose to first focus on the suppressor genes that were conserved but poorly characterized in any system, or were conserved but uncharacterized during early C. elegans embryogenesis. Prior dynein immunocytochemistry-based localization studies serve as a comparison [6,10,35]. As in other species, C. elegans DHC-1 is associated with mitotic spindles, centrosomes, the nuclear envelope, the cell cortex, the midbody, and throughout the cytoplasm. Most of the suppressor proteins we examined localized to sites where DHC-1 is known to act or localize (Figure 6). However, the nearly ubiquitous distribution of DHC-1 in early embryonic cells makes colocalization likely but not necessarily meaningful, and biochemical studies are needed to conclusively address any direct or indirect physical associations.

**Figure 7:**
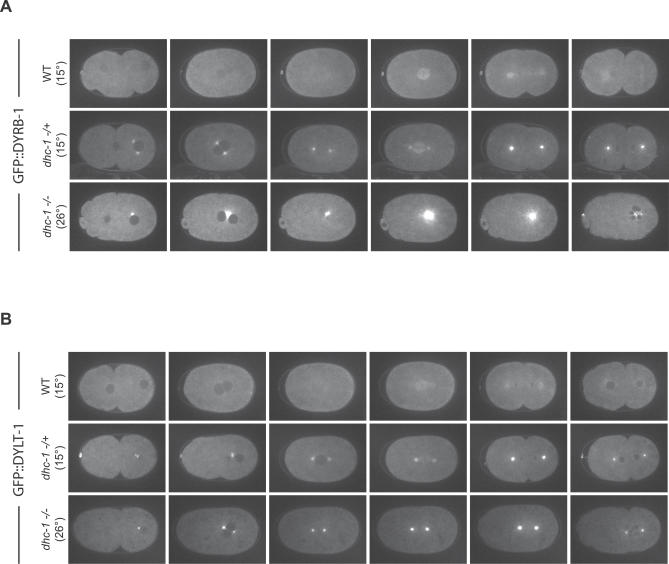
Time-Lapse Images of GFP::DYRB-1 and GFP::DYLT-1 in Wild-Type and *dhc-1(or195)* Mutant Embryos Images represent pronuclear migration to telophase in the first embryonic cell cycle. Identical conditions were used during microscopy and image manipulation so that images are directly comparable. (A) Faint localization of GFP::DYRB-1 to the spindle in a wild-type embryo (top row of images), bright labeling of centrosomes and spindle poles in an embryo from a *dhc-1(or195)* heterozygous mutant worm grown at 15 °C (middle image sequence), and very strong labeling of centrosomes and this monopolar spindle in an embryo from a *dhc-1(or195)* homozygous mutant worm shifted to 26 °C for three h (bottom row). (B) Faint localization of GFP::DYLT-1 to the spindle in a wild-type embryo (top row of images), bright labeling of centrosomes and spindle poles in an embryo from a *dhc-1(or195)* heterozygous mutant worm grown at 15 °C (middle image sequence), and very strong labeling of centrosomes and spindle poles in an embryo from a *dhc-1(or195)* homozygous mutant worm shifted to 26 °C for five h (bottom row).

Four suppressor GFP fusion proteins localized to nuclear membranes and to spindle poles or pericentrosomal regions. The DYLT-1 and DYRB-1 dynein light chains were associated with nuclear envelopes and centrosomes, as well as meiotic and mitotic spindle poles (Figure 6A–6H; Videos S1 and S2). The potential coiled-coil protein K04F10.3 was present on the nuclear envelope and in a pericentrosomal position during mitosis, similar to endoplasmic reticulum proteins [36] (Figure 6I–6L; Video S3). K04F10.3 was also highly enriched at meiotic spindle poles (Figure 6I), which has been observed for other endoplasmic reticulum proteins [36]. The NPP-22 transmembrane nucleoporin was found at nuclear envelopes (Figure 6M–6P; Video S4), as previously reported for later stage embryos [37], and it also surrounded centrosomes during mitosis. Two splice isoforms of the pleckstrin homology domain–containing EFA-6/Y55D9A.1, an ARF guanine nucleotide exchange factor, were enriched cortically both in the anterior portion of the one-cell zygote and at the blastomere boundary in two-cell embryos (Figure 6Q–6T; Videos S5 and S6). The conserved Mo25 homolog MOP-25.2/Y53C12A.4 was found enriched in a single spot after cytokinesis that appears to correspond to the midbody (Figure 6U–6X; Video S7). F10E7.8, a highly conserved ortholog of S. cerevisiae Far11, appears nuclear (Figure 6Y–6B′ and Video S8). Finally, the nonspecific suppressor protein STAR-2, a predicted RNA binding protein, appears to be associated with P-granules (like its homolog GLD-1), where dynein is neither localized nor known to function (Figure 6C′–6F′).

### DLYT-1 and DYRB-1: Dynein Light Chain Localization

The C. elegans dynein HC protein weakly localizes to spindle poles during early embryonic cell cycles [6,10], and so did DYRB-1 and DYLT-1 (Videos S9 and S10). However, ts mutant forms of the DHC-1 protein (including DHC-1 encoded by the *or195* allele) strongly localize to centrosomes when shifted to the non-permissive temperature [10]. The mechanism underlying this enhanced localization is not known, but it may represent trapping of the defective protein at a normally transient location. We exploited this behavior of the mutant DHC-1 protein to determine whether redistribution of the putative DYRB-1 and DYLT-1 dynein light chains also occurred in the *dhc-1(or195)* background.

We found that the cellular distributions of DYRB-1 and DYLT-1 were dramatically altered in *dhc-1(or195)* mutant embryos. After shifting the parental worms to the restrictive temperature for 3–5 h prior to collecting embryos, these two proteins were prominently localized to centrosomes and to spindle poles that did not separate in one-cell stage embryos (Figure 7; Videos S11 and S12). The spindle pole to cytoplasmic fluorescence ratio during late anaphase was 5-fold higher in both of the *dhc-1* homozygous mutant strains when compared to wild-type embryos expressing the GFP fusions. We also assayed localization of the two putative dynein light chains after short temperature shifts to the nonpermissive temperature in the *dhc-1(or195)* mutant background, which yields mitotic spindles with an overall wild-type appearance and function. These short temperature shifts also resulted in robust localization of these two dynein light chains to centrosomes (unpublished data). Finally, we examined the localization of GFP::DYRB-1 and GFP::DYLT-1 in embryos from *dhc-1(or195)* −/+ worms grown at the *dhc-1(or195)* permissive temperature of 15 °C. Even though embryos from mothers heterozygous for this recessive mutation are viable and develop normally, even at 26 °C [7], we observed a substantial increase in both GFP fusion proteins at the mitotic spindle poles in early embryos (Figure 7; Videos S13 and S14). Importantly, localization of DYLT-1 and DYRB-1 to centrosomes does not occur in embryos depleted for DHC-1 with RNAi (our unpublished results), indicating that these proteins require the mutant DHC-1 polypeptide for centrosomal targeting in the *dhc-1(or195)* embryos. In summary, the DYRB-1 and DYLT-1 proteins localize to sites where the DHC-1 HC is also found in wild-type embryos, and all three proteins respond similarly to mutational alterations in DHC-1.

### Genetic Characterization of the DYLT-1 and DYRB-1 Dynein Light Chains

**Figure 8:**
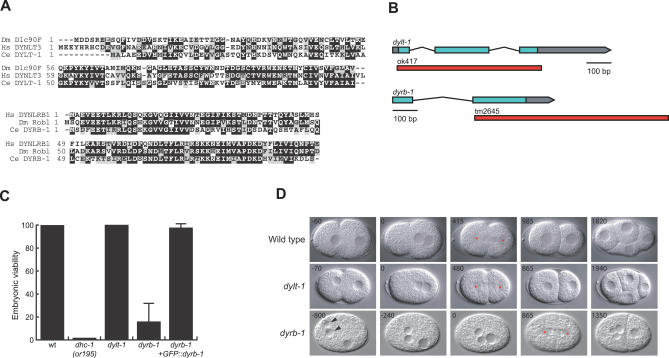
Characterization of Genes Encoding the *dylt-1* and *dyrb-1* Dynein Light Chains (A) Alignment of DYLT-1 and DYRB-1 with the homologous human and *Drosophila* proteins. The following proteins were used to make the alignments: *Dlc90F*, *DYNLT3*, *DYNLRB1*, and *robl*. (B) The genomic loci for *dylt-1* and *dyrb-1* are shown along with the locations of the deletions. Blue boxes represent exons, grey boxes are untranslated regions, and the deleted DNA is shown in red. Scale bar, in base pairs, is shown. (C) Embryonic viability observed for the dynein heavy chain mutant shifted to 23 °C, and for wild type and the dynein light chain deletion mutants cultured at room temperature. We counted the following numbers of progeny for each strain: N2: 831, *dhc-1(or195)*: 1539, *dylt-1(ok417)*: 404, *dyrb-1(tm2645)*: 273, and *dyrb-1(2645)* +*GFP::dyrb-1*: 366. (D) Time-lapse Nomarski images for wild-type, *dylt-1,* and *dyrb-1* embryos grown at room temperature. The *dylt-1* embryo displays no obvious defects, but the *dyrb-1* embryo exhibits failures in female meiotic divisions and pronuclear migration, and progression through mitosis is delayed. The time (in seconds) relative to pronuclear meeting is displayed in each panel. Red dots denote positions of spindle poles and the arrowheads point to extra maternal pronuclei in the *dyrb-1* embryo.

We obtained putative null alleles to determine if *dylt-1* and *dyrb-1* function in dynein-dependent processes. DYLT-1 is 40% identical to human DYNLT3 and 38% identical to *Drosophila* Dlc90F (see alignment in Figure 8A). Two other *C. elegans* genes, *dylt-2* and *dylt-3,* encode more divergent members of this protein family. DYRB-1 is 49% identical to both human DYNLRB1 and *Drosophila* Robl (see alignment in Figure 8A). There do not appear to be other Roadblock genes in the C. elegans genome [3]. Deletion alleles for both *dylt-1* and *dyrb-1* have been isolated (Figure 8B). The *dylt-1(ok417)* deletion removes the entire DYLT-1 open reading frame and does not affect adjacent coding regions. The *dyrb-1(tm2645)* deletion removes 69% of the *dyrb-1* coding region, leaving 29 predicted N-terminal amino acids, and does not affect adjacent coding regions.

Both deletions are currently annotated as homozygous viable [29]. However, we found that the *dyrb-1(tm2645)* strain was in fact heterozygous for the deletion and that most embryos produced by *dyrb-1(tm2645)* homozygous animals failed to hatch (Figure 8C). Homozygous *dyrb-1(tm2645)* worms also showed an egg-laying defect and produced small broods (unpublished data). To determine if the *dyrb-1* deletion was responsible for the embryonic lethality, we crossed the *GFP::dyrb-1* transgene into the deletion background. The presence of the transgene fully rescued the embryonic lethality (Figure 8C), but not the egg-laying defect: the transgene is driven from a germline-specific promoter and so would not be expected to rescue zygotic phenotypes. The embryonic lethality exhibited by *dyrb-1(tm2645)* mutants is consistent with RNAi studies performed by injection or soaking [12,38]. In contrast, homozygous *dylt-1* deletion mutants did not exhibit any larval or embryonic lethality (Figure 8C).

To determine if these dynein light chain mutants exhibit dynein HC-like phenotypes, we observed the completion of meiotic polar body extrusion and the first two mitotic cell divisions in mutant embryos (Figure 8D). The *dylt-1* embryos appeared wild type for completion of meiosis, pronuclear migration, and spindle assembly and function. However, the *dyrb-1* embryos occasionally contained extra female pronuclei (observed in four of 12 recordings, Figure 8D), suggesting that polar body extrusion was defective, and pronuclear migration was often slow compared to wild-type embryos. Once formed, spindles appeared functional using Nomarski optics, although they were frequently positioned improperly and had large spindle poles, as has also been observed after RNAi knockdown [12]. Thus, these two genes are not strictly essential, but the DYRB-1 protein clearly is required for dynein-dependent processes.

## Discussion

By using the suppressor screening method outlined in Figure 2B, we have isolated and characterized 49 genes that when reduced in function can suppress a partial loss of dynein HC function. This screening procedure takes advantage of sensitized genetic backgrounds using conditional mutants, can be completed for one mutant in less than 5 wk, and is scalable so that many mutants can be screened in parallel. In fact, we have performed 15 such screens in different sensitized backgrounds (unpublished data). By using three *dhc-1*ts mutant strains, we found that strain background differences and allele specificity are minimal because most of these genes suppress all three dynein mutants when reduced in function using RNAi. Furthermore, by using two unrelated ts mutants to assay for specificity, we found that 57% of the suppressor genes suppress multiple unrelated mutant loci. Thus, it is clear that assaying the specificity of suppression is critical for evaluating the functional significance of these RNAi interactions. Eliminating the analysis of these nonspecific genes in future screens will save time and resources. Most of the specific suppressor proteins we examined appear to overlap in subcellular localization with the dynein HC, based on previous studies of DHC-1, while one nonspecific suppressor protein, STAR-2, localized to germline P-granules, where dynein is not known to function.

Many of the 20 genes that specifically suppress multiple *dhc-1*ts alleles are nonessential in C. elegans but well conserved nonetheless. Six of eight deletion alleles available for the 20 specific suppressor genes are homozygous viable, and six additional specific genes do not display lethal phenotypes when reduced in function by RNAi in wild-type worms [29]. Thus, our genetic screening has identified roles in an essential process for at least 12 apparently nonessential genes. Fourteen of the specific dynein suppressor genes have human orthologs as determined by best reciprocal BLAST hits (Table 1), while *mop-25.2* has a conserved human homolog but also a paralog in C. elegans. Eleven of these conserved genes are nonessential in C. elegans. Interestingly, eight of the conserved genes in Table 1 have been implicated in human disease etiology, with three of them identified as the causative gene [39–41]. Thus, using sensitized genetic backgrounds for genome-wide modifier screens can identify roles for nonessential but conserved genes and thereby provide insights into human disease.

**Table 1:**
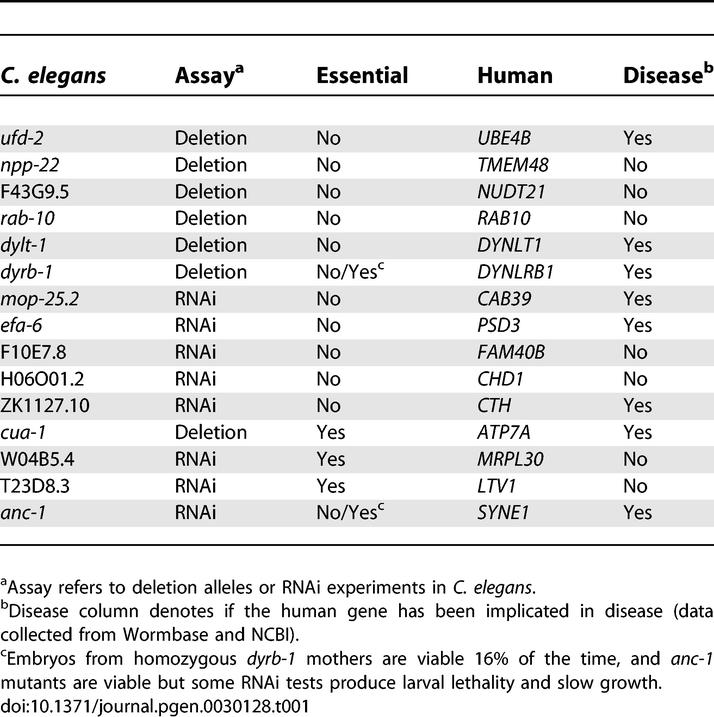
Conserved and Specific *dhc-1* Suppressor Genes

### Nonspecific Suppression of Conditional Mutants

We examined the predicted molecular functions of the suppressor proteins to better understand the basis for the nonspecific suppression phenomenon. Strikingly, many of the nonspecific suppressor genes encode proteins with predicted roles in mitochondrial, ribosomal, and collagen function (18 of 29 genes, or 62%), while only two such genes appeared to specifically suppress *dhc-1* (2 of 20 genes or 10%). It is possible that stress produced by RNAi knockdown of these suppressor genes triggers the activity of molecular chaperones that can generally restore function to ts proteins. Indeed, mutation of *dpy-10* is known to suppress three other ts mutants: *glp-1*, *emb-5,* and *mup-1* [42–44]. Furthermore, RNAi reduction of *dpy-10*, *star-2*, *osr-1,* or C50D2.1 (all suppressors of *dhc-1, lit-1, and spn-4* ts alleles) induces the glycerol biosynthetic gene *gpdh-2,* while *dpy-10* and *osr-1* mutants exhibit increased glycerol levels, a condition that promotes protein stability [45]. We suggest that partial loss of central metabolic processes can invoke stress responses that nonspecifically alleviate protein-folding problems in ts proteins. Filtering out these nonspecific interactions by testing unrelated conditional mutants increases the likelihood that the remaining suppressor genes are more directly involved with dynein function. However, ts mutants likely differ in their susceptibility to nonspecific suppression mechanisms, and some apparently unrelated ts mutants might share common cofactors such that both mutants are suppressed by depletion of the same cofactor. Nevertheless, we expect that more extensive testing for specificity will prove very useful for judging the significance of modifier interactions.

### Possible Relevance of Suppressor Proteins to Dynein Function

We examined the localization of a number of GFP fusions to suppressor proteins to gain insight into their functional relationship to dynein. In several cases, the subcellular distribution of the suppressor proteins overlapped in different ways with the known and nearly ubiquitous distribution of cytoplasmic dynein in the early C. elegans embryo. In fact, the only specific suppressor that did not display dynein-like localization was F10E7.8, a homolog of yeast Far11 of unknown function [46], which was nuclear. The one nonspecific suppressor protein we examined did not show any dynein-like localization patterns. The subcellular localizations of the GFP::suppressor protein fusions are intriguing. However, given the nearly ubiquitous distribution of dynein in early embryonic cells, biochemical tests for direct association are needed to address the significance of any colocalization detected using light microscopy.

We are particularly interested in suppressor proteins that localize to mitotic spindle poles: the association of the DYLT-1 and DYRB-1 predicted dynein light chains with centrosomes and spindle poles suggests that they may be components of cytoplasmic dynein in C. elegans. Localization of cytoplasmic dynein to centrosomes and spindle poles is well established [47,48], and the inhibition of dynein function prevents centrosome separation, centrosome attachment to nuclei, and the formation of bipolar spindles [6,10,49,50]. Moreover, the centrosomal localization of DHC-1, DYLT-1, and DYRB-1 are all greatly enhanced in *dhc-1*ts mutant embryos: this dependence of the light chain distribution on the HC further suggests they reside in the same motor complex (Figure 7 and [10]). Furthermore, roadblock light chains are well-established components of dynein, and all of the roadblock protein in mammalian liver extracts is dynein associated [51,52]. Finally, a DYLT-1 homolog in vertebrates is a stoichiometric subunit of cytoplasmic dynein [53]. The presence of these two light chains in a dynein complex is consistent with them having either positive or negative roles in the regulation of HC function (see below).

Cytoplasmic dynein is found on the nuclear envelope where it is thought to regulate nuclear membrane breakdown during mitosis [54], and dynein plays roles during the trafficking of endoplasmic reticulum components [55,56]. Therefore, the nuclear envelope/endoplasmic reticulum proteins NPP-22 and K04F10.3 could couple dynein activity to either of these structures. The *anc-1* gene was also isolated in our screening and ANC-1 is localized to the nuclear envelope where it maintains nuclear positioning in postembryonic cells [57]. Reducing the function of these three genes may suppress partial loss of dynein HC mutants by reducing the need for dynein during nuclear envelope breakdown, through constitutive partial destabilization of the nuclear envelope.

The distribution of the cytoplasmic dynein HC includes sites other than spindle poles and nuclear envelopes in *C. elegans,* for example, at the cell cortex and at the cell division remnant called the midbody [6,10]. The MOP-25.2 protein was found at the midbody and faintly at spindle poles. The MOP-25.2 ortholog in *S. pombe,* Pmo25, is also present at the cell division site and on spindle poles [58]. Mammalian MOP-25.2 homologs stimulate the kinase activity of the LKB1 tumor suppressor (the C. elegans ortholog is PAR-4), which in turn activates MARK microtubule-destabilizing kinases [59,60]. The C. elegans MARK ortholog, PAR-1, controls cell polarity during embryogenesis, and orthologs have been implicated in regulation of microtubule dynamics from yeast to humans [61–64].

Lastly, the two splice isoforms of EFA-6 were associated with the anterior cell cortex in late one-cell embryos. Cortically localized dynein may have important roles in applying forces to astral microtubules that influence mitotic spindle positioning and chromosome separation during anaphase [10,65,66]. EFA6 ARF guanine nucleotide exchange factors require their pleckstrin homology domain for cortical targeting, and are known to regulate cortical actin dynamics in vertebrate cells by promoting guanine nucleotide exchange on ARF6 [67,68]. Our results identifying *efa-6* as a dynein HC suppressor suggest a functional linkage of the actin and microtubule cytoskeletons at the cell cortex. Interestingly, two yeast pleckstrin homology domain proteins, Num1 and mcp5+, localize to the cell cortex and direct astral microtubule and dynein function, although they do not contain a Sec7 domain like EFA-6 does [69–71].

### Function of Dynein Intermediate, Light Intermediate, and Light Chains

The dynein chains in C. elegans exhibit strikingly different functional requirements. The DYRB-1 roadblock light chain is required for completion of meiosis and pronuclear migration, but an at least partially functional mitotic spindle forms in the absence of DYRB-1 (Figure 8). The DLI-1 light intermediate chain is required for multiple dynein-dependent functions: pronuclear migration, centrosome separation, and meiotic and mitotic spindle function [12,34]. DLI-1 may promote nuclear envelope targeting of both centrosomes and DHC-1 by interacting with the nuclear envelope protein ZYG-12 [35]. The second worm light intermediate chain gene, *xbx-1,* is required for cilia function but not early embryonic development [12,72]. RNAi knockdown of DLC-1, one of three LC8 proteins in *C. elegans,* produces defects similar to *dli-1* but knockdown of the other two LC8-related genes does not result in any phenotypes [12]. RNAi depletion of *dyci-1* results in severe meiotic, pronuclear migration, and mitotic spindle assembly defects [12] and in our feeding RNAi regimen *dyci-1(RNAi)* produces a larval arrest phenotype similar to that observed for *dhc-1*. Finally, the three Tctex1 proteins in *C. elegans,* DYLT-1, 2, and 3, are not essential for dynein-related functions [12]. As the *dyrb-1, dlc-1,* and *dli-1* dynein AC genes display some *dhc-1*-like requirements, they positively influence dynein function. However, because reducing their function suppresses *dhc-1*ts mutants, they may also exert negative regulation (along with *dylt-1*) on the heavy chain.

### Negative Regulation of Dynein HC by Light Chain Subunits

Finding that reducing the function of light and light intermediate dynein chains suppressed the partial loss of HC function was a striking result. One member of each of four subunit classes can suppress the embryonic lethality associated with three *dhc-1* ts mutants (Figure 5). We have considered two different models to explain how RNAi-mediated depletion of these dynein ACs can suppress reduced HC function. First, these dynein subunits could be in functional complexes with, and exert negative regulation on, the DHC-1 HC (Figure 5B). The suppression mechanism in this case proposes that removal of the suppressing ACs increases residual mutant DHC-1 activity. The other, nonsuppressing, accessory subunits might then function in nonmitotic cellular processes such as neuronal transport or organelle positioning. In support of this view, physical removal of the intermediate chains of rat cytoplasmic dynein increased HC ATPase activity by about 4-fold (light chains were not monitored in this study but were likely removed as well) [73]. Thus, at least with respect to ATPase activity, some dynein ACs do act as biochemical negative regulators of HC function.

### An Assortment of Essential and Nonessential Dynein Complexes

Alternatively, an assortment of dynein complexes (with different ACs) could coexist within early embryonic cells, with only a subset required for the essential mitotic functions that require DHC-1. In this case, suppression might result from the release of DHC-1 HCs from less essential motor complexes, allowing more of the functionally compromised HCs to participate in the essential process of mitosis. We currently disfavor this hypothesis because two of the suppressing light chains (DYLT-1 and DYRB-1) can indeed localize to meiotic and mitotic spindles (Figures 6 and 7), sites where DHC-1 has been shown by others to localize and function. Furthermore, the distribution of DYRB-1 and DYLT-1 closely resembles the distribution of the HC in *dhc-1(or195)* embryos, suggesting that these two light chains associate with the HC during mitosis (Figure 7 and [10]). Finally, *dhc-1*-like phenotypes result from mutation or RNAi knockdown of three suppressing ACs in otherwise wild-type worms, indicating that they share at least some common and essential requirements. Regardless of the suppression mechanism, our identification of ACs that genetically interact with the DHC-1 HC provides a basis for functionally classifying the paralogs of these dynein subunit gene families, and for further investigation of dynein composition and function.

### Nonessential Dynein Subunits and Negative Regulation of the HC

Some ACs are nonessential, supporting the view that some cytoplasmic dynein subunits could function by exerting negative regulation on the HC, rather than positively influencing essential HC function. For example, DYRB-1 is not absolutely required for viability because worms lacking this protein can be propagated, although they are extremely sick and do exhibit two *dhc-1*-like phenotypes (Figure 8). Also, homozygous *dylt-1* deletion mutants appear fully viable (Figure 8). The two additional Tctex1 C. elegans genes could be functionally redundant with DYLT-1, but simultaneously reducing the function of DYLT-2 and DYLT-3 by RNAi in the *dylt-1* deletion strain did not cause lethality (unpublished data). Because RNAi does not always completely reduce function, the question of redundancy in the Tctex1 C. elegans gene family remains unresolved. However, *Drosophila* contains only a single Tctex1 gene, *Dlc90F* [74,75]. A *Dlc90F* null allele that deletes 80% of the open reading frame is essential only for sperm production but not for viability of male or female flies, despite the wild-type protein being incorporated into dynein motors and expressed in various *Drosophila* tissues [74]. Thus, at least in *Drosophila,* the Tctex1 dynein light chain family is not required for cell division processes like the HC is. Interestingly, budding yeast does not possess genes for the Tctex1 or roadblock ACs, indicating that functional cytoplasmic dynein does not require these subunits that are conserved in many other organisms. The AC genes that yeast does posses are not required for HC motility in vitro because dynein purified from yeast with mutations in these genes remains fully active [2]. Thus, dynein function in several contexts does not require AC subunits, and we suggest that in some cases they may have negative regulatory roles. Negative regulation of cytoplasmic dynein may be redundant with other modes of HC regulation or only required during special circumstances. Further studies of subunit localization, and in vitro studies of C. elegans dynein motility, may provide further insight into the modes of AC regulation and function.

## Materials and Methods

### C. elegans strains and culture.

Strains were cultured according to standard procedures [28]. ts mutants were maintained at 15 °C and GFP-expressing strains in a wild-type background were maintained in a 23 °C incubator. *dhc-1(or195)* was outcrossed six times to the N2 Bristol wild-type strain and the *or283* and *or352 dhc-1* mutants were each outcrossed four times with N2. For sequencing mutant *dhc-1* loci, genomic DNA was amplified as overlapping ∼1-kb fragments from the start codon to the stop codon and sequenced at the University of Oregon DNA sequencing laboratory. For double mutant constructions, the *dhc-1(or195)* mutation was monitored by sequencing or by assaying a restriction fragment length polymorphism caused by the mutation with Hpy188I (New England Biolabs, http://www.neb.com), following PCR amplification of the mutated region. The *dylt-1(ok417)* and *ufd-2(tm1380)* alleles were monitored by PCR amplification of genomic sequence encompassing the deletions and assaying product size by agarose gel electrophoresis.

### GFP imaging.

Visualization of GFP fusion protein localization was accomplished by mounting embryos on M9 + 3% agarose pads on microscope slides covered with a coverslip. Time-lapse videos were obtained on a spinning disk Nikon Eclipse TE2000-U microscope (Nikon Instruments, http://www.nikon.com) fitted with an ORCA-ER digital camera (Hamamatsu Photonics, http://www.hamamatsu.com) using a Nikon 60×, 1.4 NA Plan Apo oil objective lens. Videos were adjusted for contrast in ImageJ (National Institutes of Health, http://rsb.info.nih.gov/ij/) [76], images were adjusted for levels in Adobe Photoshop (http://www.adobe.com/).

### RNAi screening.

After obtaining the E. coli RNAi library from the MRC Geneservice (Cambridge, UK) [17,19], we rearrayed it into a 48-well microplate format using a liquid-handling Qiagen BioRobot 8000 (http://www.qiagen.com). E. coli strains were thawed from −80 °C storage and inoculated into 1 ml of LB + 100 mg/ml ampicillin-containing 96-well growth plates (Whatman, http://www.whatman.com) and covered with microporous sealing film (USA Scientific, http://www.usascientific.com). Only 48 wells of the 96-well growth plates were filled with media, corresponding to the rearrayed E. coli library. After overnight shaking incubation at 37 °C, 20 μl of the cultures were dispensed with a 24-channel electronic repeating pipette (Rainin, http://www.rainin.com) onto 48-well plates (Nunc, http://www.nuncbrand.com/) containing NGM agar, 100 μg/ml ampicillin, and 1 mM IPTG and allowed to dry and induce dsRNA at 37 °C overnight. The 48-well agar plates were filled with a Wheaton Unispense peristaltic pump (http://www.wheaton.com) equipped with a custom-made adaptor (University of Oregon Technical Science Administration) that allowed simultaneous filling of eight wells with the agar solution. Approximately 15 hypochlorite-synchronized L1 mutant larvae were pipetted into each well of the 48-well plates with a multichannel pipette and allowed to produce broods. Screening for F1 viability was performed by visual examination with a dissecting microscope. Phenotypes were recorded on an Excel spreadsheet (http://www.microsoft.com) and organized in a FileMaker Pro (http://www.filemaker.com) database. We qualitatively identified 295 initial positive suppressing E. coli strains, for which we repeated the assay on 60-mm plates with E. coli again thawed from the library (not streak purified). If the observed phenotypes reproduced, the assay was performed with three streak-purified E. coli colonies, and a single isolate that again displayed the interaction was kept for further analysis.

### Embryonic viability quantitation methods.

To quantitate embryonic viability we used the following procedure. Cultures of dsRNA-producing bacteria were grown overnight in LB + 100 μg/ml ampicillin. Cultures (0.2 ml) were seeded onto 60-mm NGM agar plates containing 100 μg/ml ampicillin and 1 mM IPTG and allowed to induce dsRNAs overnight at room temperature. The L4440 control vector-containing strain was used as the bacterial lawn for the experiments shown in Figures 2A and 3B. Approximately 80 synchronized L1 larvae (obtained from hypochlorite-treated worms) were pipetted onto the plates and allowed to grow to young adulthood. Five gravid worms were transferred to prepared NGM agar plates supplemented with 100 μg/ml ampicillin and 1 mM IPTG containing a small RNAi bacterial lawn produced from ∼5 μl of overnight E. coli culture. After producing broods, the adult worms were removed and the embryos were allowed to develop for at least 24 h. Embryos and larvae were then counted immediately or after storage at 4 °C. We considered only suppressor dsRNAs that increased embryonic viability greater than 3-fold above the background viability observed with the L4440 control vector (in the *dhc-1(or195)*) screen to be significant enough for continued study.

### Molecular biology.

We introduced a polylinker site containing six unique restriction enzyme recognition sites into the pIC26 GFP-S protein plasmid by using phosphorylated and PAGE-purified synthetic oligonucleotides [77]. Following ligation, the new plasmid was sequence verified. The modified vector, pSO26, allows the use of additional restriction enzymes and directional cloning for inserting genes of interest (Figure S2). The SpeI site was recreated at the 5′ end of the polylinker but not at the 3′ end. We amplified N2 genomic DNA or cDNA (Invitrogen, http://www.invitrogen.com) with Pfu Turbo polymerase (Stratagene, http://www.stratagene.com), and cloned A-tailed PCR products into either pGEM-T or pGEM-T-easy shuttle vectors (Promega, http://www.promega.com). Inserted genes were sequence verified at the University of Oregon DNA sequencing laboratory prior to cleavage and ligation to pSO26 (see Table S3 for restriction sites and primer sequences used). All of the constructs used in this study were cloned as SpeI-AsiSI or AscI-AsiSI fragments, except for the STAR-2 gene, which was amplified as a SpeI fragment and cloned into pIC26.

To construct dynein subunit dsRNA-expressing plasmids not available in the RNAi library, gene fragments were amplified from N2 genomic DNA with the following primers: F41G4.1: 5′-AAGATATCACCCAAAATGGTCCAAAACAAAG-3′ and 5′-CGGATATCTCGACTGAAGCTGGTTCTGA-3′, *xbx-1:* 5′-AAGATATCTACGACGATGGAAGTTTGAAG-3′ and 5′-CGGATATCCGTGCCTCTGCAGC-3′, *dlc-3:* 5′-AAGATATCAATTTCAGGTGGACACTGGC-3′ and 5′-CGGATATCAGCACACTTGCATCATCTGAA-3′. The PCR products were cut with EcoRV, ligated to EcoRV-digested L4440, and sequence verified.

### Isolation of transgenic worms.

GFP fusion plasmids were bombarded into *unc-119(ed3)* worms as previously described except with the following two changes [78]. Three milligrams of gold particles were used per hepta adaptor bombardment. Also, we briefly sonicated the gold particles (prior to DNA coating and while suspended in 50% glycerol) with a Branson sonifier 450 (http://www.sonifier.com/) fitted with a small tip set to power level 1, to disrupt gold aggregates. Non-Unc worms were picked to new plates and allowed to produce broods, which were assayed for GFP fluorescence with a Zeiss axioskop microscope (http://www.zeiss.com/) fitted with an X-Cite 120 illumination system (EXFO life sciences, Mississauga, Ontario, Canada). For each fluorescent line, 12 GFP-positive worms were singled to new plates to determine if the constructs were integrated or were carried as extrachromosomal arrays.

## Supporting Information

Figure S1 Suppression of *dhc-1* Early Embryonic Defects Twenty-three representative suppressor genes were tested for their effect on spindle length and cytokinesis in *dhc-1* embryos. (A) Wild-type, *dhc-1(or195),* and *dhc-1(or195)*; *dylt-1(RNAi)* embryos at early telophase and subsequent to cytokinesis. Reduction of *dylt-1* function in the *dhc-1* strain partially restores spindle length and cytokinesis is successful. (B) Graph of P_0_ spindle length and cytokinesis success for *dhc-1(or195)* embryos with reduced suppressor gene function (values expressed as percent of wild type). The numbers in parentheses denote the number of embryos recorded for each gene depleted with RNAi. The L4440 trial represents *dhc-1(or195)* animals feeding on bacteria harboring the empty RNAi vector. Genes were separated into specific and nonspecific suppressors based on Figure 4, and are sorted by spindle lengths.
(1.6 MB PDF)

Figure S2 Sequence of the New Multiple Cloning Site in pSO26 The two oligonucleotides shown were ligated into SpeI-cut pIC26 to yield pSO26. pIC26 uses the *pie-1* promoter and 3′ UTR and contains the *unc-119*(+) transformation marker [77]. Unique restriction sites are shown, enzymes giving blunt ends are indicated with a “b,” and enzymes with 8-base recognition sites are shown with an “8.” The translational reading frame is represented as codon triplets. The SpeI site was recreated and remains unique.
(75 KB PDF)

Table S1 Quantification of Embryonic Viability in RNAi-Treated ts Mutants Data was used to prepare Figure 3A and Figure 4. Number of progeny counted, embryonic viability, and embryonic viability standard deviation is shown for each RNAi assay. Numbers of progeny are not comparable. The rightmost four columns summarize information from other parts of this paper, and two dsRNAs are predicted to knock down expression of paralogous genes.
(27 KB XLS)

Table S2 Quantification of Embryonic Viability in RNAi-Treated *dhc-1*ts Mutants Data was used to prepare Figure 5A. Number of progeny counted, embryonic viability, and embryonic viability standard deviation is shown for each RNAi assay. Numbers of progeny are not comparable.
(20 KB XLS)

Table S3 GFP Fusion Protein Information PCR template type, cloning sites, primer sequences, and a representative C. elegans line are given for each gene.
(23 KB XLS)

Video S1 GFP::DYLT-1, Localization in Two Gastrulation-Stage Embryos Localization to nuclear envelopes and centrosomes. Three slices 1 μm apart were captured every 30 s and projected.
(1.0 MB MOV)

Video S2 GFP::DYRB-1, Localization to Meiotic Spindle Poles during Meiosis I and II Three slices 1 μm apart were captured every 30 s and projected.
(709 KB MOV)

Video S3 GFP::K04F10.3, nuclear membrane and pericentrosomal localization (similar to endoplasmic reticulum proteins) Three slices 1 μm apart were captured every 30 s and projected.
(1.5 MB MOV)

Video S4 GFP::NPP-22, Nuclear Membrane and Pericentrosomal Localization (Similar to Endoplasmic Reticulum Proteins) Note the appearance of “strings” in the nucleus that might represent furrows in the nuclear envelope. One slice was captured every 30 s.
(920 KB MOV)

Video S5 GFP::EFA-6.a, Localization to the Anterior Cortex in the One-Cell Embryo and Enrichment to the Boundary between the AB and P1 Cells Not present in four-cell embryos. Midbody localization is seen at 30 min and a polar body is seen in the 31–34.5 min frames. One slice was captured every 30 s.
(768 KB MOV)

Video S6 GFP::EFA-6.c, Similar to EFA-6.a (Video S5) One slice was captured every 30 s.
(678 KB MOV)

Video S7 GFP::MOP-25.2, Localization to the Midbody and Weakly Nuclear Three slices 1 μm apart were captured every 30 s and projected.
(1.5 MB MOV)

Video S8 GFP::F10E7.8, Nuclear Localization The cytoplasmic signal is partially due to background autofluorescence. One slice was captured every 30 s.
(1.4 MB MOV)

Video S9 GFP::DYRB-1 in a Wild-Type Background Worms were grown at 15 °C and embryos were imaged on a room temperature microscope stage. Weak localization to mitotic spindle poles. Three slices 1 μm apart were captured every 30 s and projected.
(342 KB MOV)

Video S10 GFP::DYLT-1 in a Wild-Type Background Worms were grown at 15 °C and embryos were imaged on a room temperature microscope stage. Weak localization to mitotic spindle poles. Three slices 1 μm apart were captured every 30 s and projected.
(249 KB MOV)

Video S11 GFP::DYRB-1 in a *dhc-1(or195ts)* Homozygous Background Strain grown at 15 °C, shifted to 26 °C for 3 h and imaged on a microscope stage maintained at 26 °C. Robust localization to centrosomes. Three slices 1 μm apart were captured every 30 s and projected.
(313 KB MOV)

Video S12 GFP::DYLT-1 in a *dhc-1(or195ts)* homozygous background Strain grown at 15°C, shifted to 26°C for 5 hours and imaged on a microscope stage maintained at 26°C. Robust localization to centrosomes. Three slices 1 μm apart were captured every 30 s and projected.
(386 KB MOV)

Video S13 GFP::DYRB-1 in a *dhc-1(or195ts)* Heterozygous Background Strain grown at 15 °C and embryos were imaged on a room temperature microscope stage. Robust localization to mitotic spindle poles. Three slices 1 μm apart were captured every 30 s and projected.
(240 KB MOV)

Video S14 GFP::DYLT-1 in a *dhc-1(or195ts)* Heterozygous Background Strain were grown at 15 °C and embryos were imaged on a room temperature microscope stage. Robust localization to mitotic spindle poles. Three slices 1 μm apart were captured every 30 s and projected.
(206 KB MOV)

### Accession Numbers

The National Center for Biotechnology Information (NCBI) database (http://www.ncbi.nlm.nih.gov/gquery/gquery.fcgi?itool=toolbar) accession numbers for the *dhc-1* homologs discussed in this paper are *C. elegans,* NP_491363; *Dictyostelium discoideum,* XP_643185; *Drosophila melanogaster,* AAA60323; *Homo sapiens,* NP_001367; *Mus musculus,* NP_084514; *Saccharomyces cerevisiae,* NP_012980; and *Schizosaccharomyces pombe,* NP_001018285.

The NCBI accession numbers for the *Drosophila* and human DYLT-1 and DYRB-1 protein homologues, respectively, are *Dlc90F*, NP_477356; *DYNLT3*, NP_006511; *DYNLRB1*, NP_054902; and *robl,* NP_523771.

## Abbreviations

AC: accessory chain
dsRNA: double-stranded RNA
GFP: green fluorescent protein
HC: heavy chain
IPTG: isopropyl-beta-D-thiogalactopyranoside
RNAi: RNA interference
ts: temperature-sensitive
