# Supplementary material for: Dynein Modifiers in C. elegans: Light Chains Suppress Conditional Heavy Chain Mutants
Source: PLoS Genet. 2007 Aug 3;3(8):e128. doi: 10.1371/journal.pgen.0030128 (PMC1937013; doi:10.1371/journal.pgen.0030128)

Figure S1, O'Rourke et al.

A

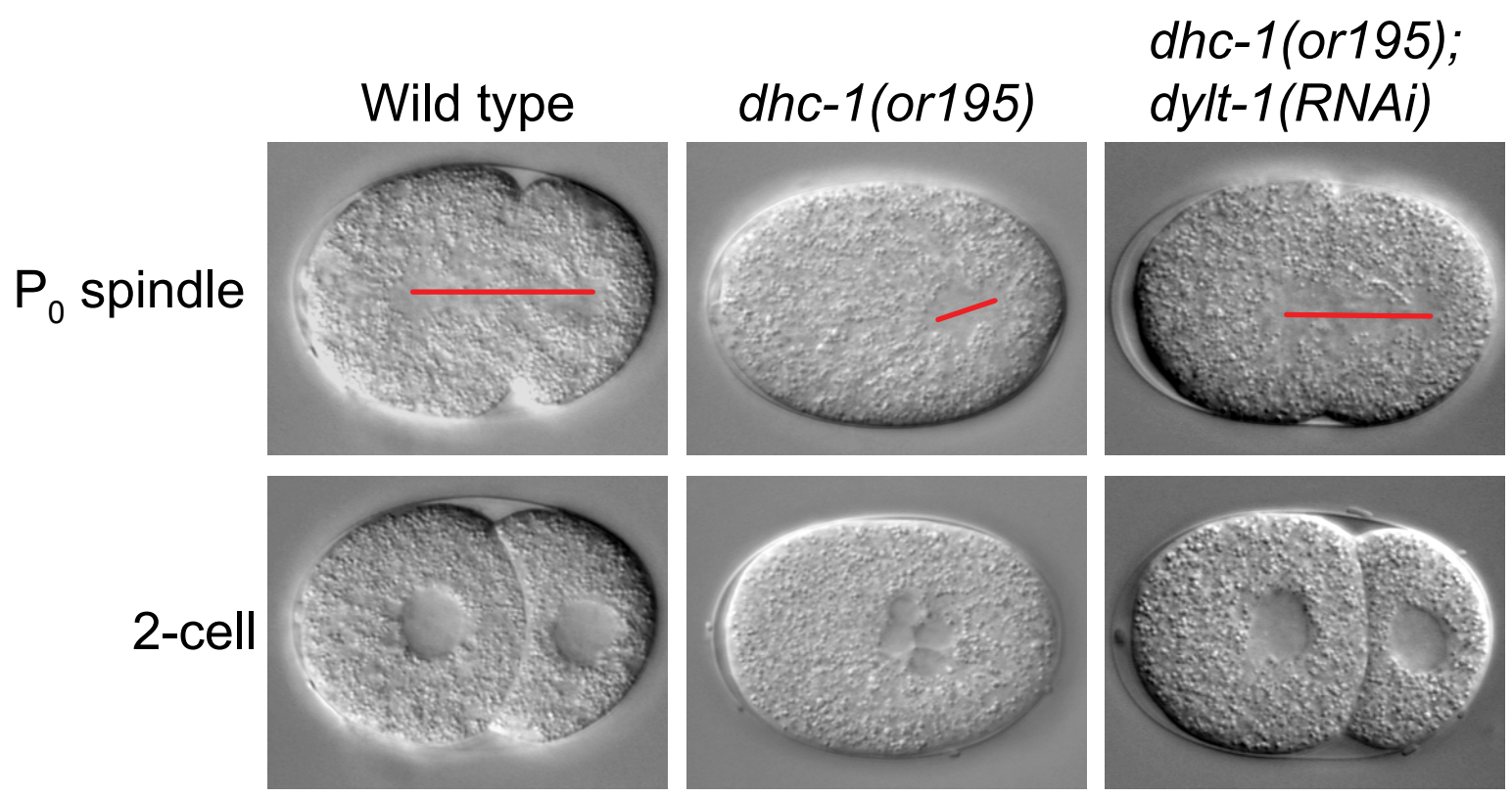

B

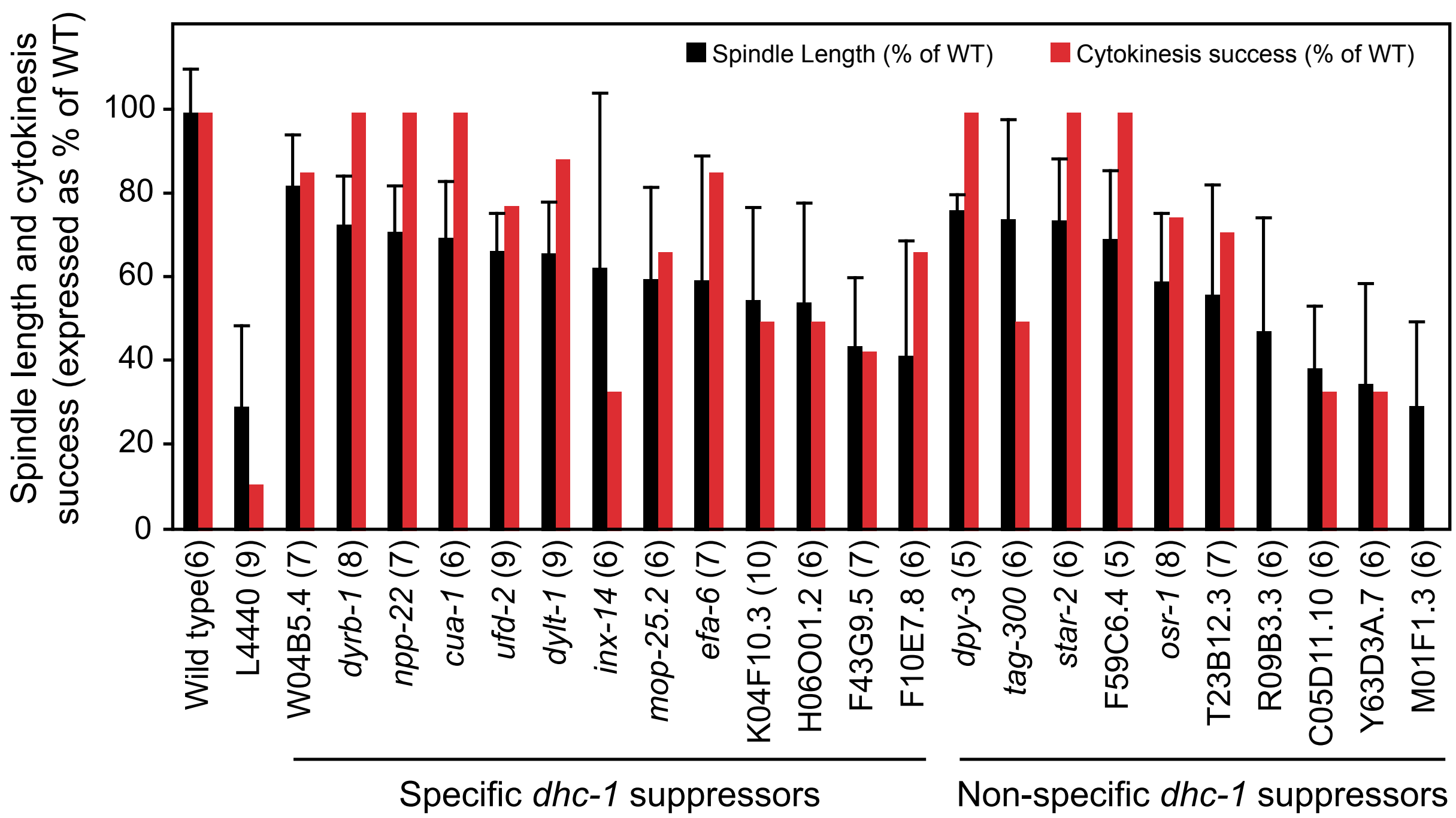

Supplement: Supplementary file 1 [file pgen.0030128.sg001.pdf]
