# Supplementary material for: Dynein Modifiers in C. elegans: Light Chains Suppress Conditional Heavy Chain Mutants
Source: PLoS Genet. 2007 Aug 3;3(8):e128. doi: 10.1371/journal.pgen.0030128 (PMC1937013; doi:10.1371/journal.pgen.0030128)
Supplement: Supplementary file 2 [file pgen.0030128.sg002.pdf]

Figure S2, O'Rourke et al.

```
CT AGT gga GGC GCG CCa gga GTT AAC gga TGG CCA tct gaG CGA TCG Cag ggA GGC CTt taa t
     A cct CCG CGC GGt cct CAA TTG cct ACC GGT aga ctC GCT AGC Gtc ccT CCG GAa att aga tc
 SpeI         AscI          HpaI        MscI          SgfI/AsiSI       StuI
               (8)           (b)         (b)             (8)            (b)
```
