# Supplementary material for: Dynein Modifiers in C. elegans: Light Chains Suppress Conditional Heavy Chain Mutants
Source: PLoS Genet. 2007 Aug 3;3(8):e128. doi: 10.1371/journal.pgen.0030128 (PMC1937013; doi:10.1371/journal.pgen.0030128)
Supplement: Supplementary file 3 [file pgen.0030128.st003.pdf]

Table S3. O'Rourke et al.
GFP fusion protein information

Gene	DNA template	cloning sites	left primer	right primer	line
npp-22	genomic DNA	AscI/AsiSI	GGAGGCGCGCCAATGATGGGTGATAGTCATTCTTCATTC	CCTGCGATCGCTTACAATTCATCTGTGAGACAAATCATCC	orIs13 [pie-1 promoter::GFP::npp-22 gDNA fusion::pie-1 3’UTR unc-119(+); unc-119(ed3)III]
K04F10.3	cDNA	AscI/AsiSI	GGAGGCGCGCCAATGTCCGATGATGAAAGTATTTTTGTTC	CCTGCGATCGCTTAGTTTGTAGAATCACCAGCAGCG	orIs12 [pie-1 promoter::GFP::K04F10.3 cDNA fusion::pie-1 3’UTR unc-119(+); unc-119(ed3)III]
dylt-1	genomic DNA	SpeI/AsiSI	GGTACTAGTATGGCTTTGGCAGAGGAGGA	CCTGCGATCGCTTAAATTGCAATTGCAAAAACATAGACG	orEx8 [pie-1 promoter::GFP::dylt-1 gDNA fusion::pie-1 3’UTR unc-119(+); unc-119(ed3)III]
dyrb-1	genomic DNA	SpeI/AsiSI	GGTACTAGTATGTCTGATTTCGAGGAGACTATTCG	CCTGCGATCGCTTACGAGAGGTCCTTGATAACCATAATG	orEx9 [pie-1 promoter::GFP::dyrb-1 gDNA fusion::pie-1 3’UTR unc-119(+); unc-119(ed3)III]
mop-25.2	genomic DNA	SpeI/AsiSI	GGTACTAGTATGTTGAAGCCGTTGTTCGG	CCTGCGATCGCTCAGACACGAAGTTCTTGTATCTGCT	orEx7 [pie-1 promoter::GFP::mop-25.2 gDNA fusion::pie-1 3’UTR unc-119(+); unc-119(ed3)III]
efa-6.a	cDNA	SpeI/AsiSI	GGTACTAGTATGGCGAAGGTGGCGTC	CCTGCGATCGCTCAATGAGCAGCAGCTCCATTA	orIs15 [pie-1 promoter::GFP::efa-6.a cDNA fusion::pie-1 3’UTR unc-119(+); unc-119(ed3)III]
efa-6.c	cDNA	SpeI/AsiSI	GGTACTAGTATGGCGAAGGTGGCGTC	CCTGCGATCGCTCAATGAGCAGCAGCTCCATTA	orIs14 [pie-1 promoter::GFP::efa-6.c cDNA fusion::pie-1 3’UTR unc-119(+); unc-119(ed3)III]
star-2	cDNA	SpeI	GGTACTAGTATGACACACGCTTCCACGGATC	CCTACTAGTTTACCTTAACAAAAGTTGTTGATATTCAGCAGCC	orIs11 [pie-1 promoter::GFP::T21G5.5 cDNA fusion::pie-1 3’UTR unc-119(+); unc-119(ed3)III]
F10E7.8	genomic DNA	SpeI/AsisI	GGTACTAGTATGCCTGCACATCTAAACGTTAAAG	CCTGCGATCGCTTACATAAGATCTTCCACTCCACGTGTC	orEx10 [pie-1 promoter::GFP::F10E7.8 gDNA fusion::pie-1 3’UTR unc-119(+); unc-119(ed3)III]
